# How Deeply Can mRNA Vaccines Affect the Responsiveness to Immune Checkpoint Inhibitors Through Changes in the Tumor Microenvironment? Evidence from Melanoma

**DOI:** 10.3390/cells15110986

**Published:** 2026-05-27

**Authors:** Ivana Persico, Maria Grazia Doro, Laura Frogheri, Maria Cristina Sini, Giovanni Battista Maestrale, Antonella Manca, Domenico Mallardo, Paolo Antonio Ascierto, Giuseppe Palmieri

**Affiliations:** 1 Unit of Cancer Genetics, Institute of Genetics and Biomedical Research (IRGB), National Research Council (CNR), 07100 Sassari, Italy; ivana.persico@cnr.it (I.P.); mariagrazia.doro@cnr.it (M.G.D.); marialaura.frogheri@cnr.it (L.F.); mariacristina.sini@cnr.it (M.C.S.); giovannibattista.maestrale@cnr.it (G.B.M.); antonella.manca@cnr.it (A.M.); 2 Istituto Nazionale Tumori (INT), Fondazione “Giovanni Pascale”, 80040 Naples, Italy; dome.mallardo@gmail.com (D.M.); paolo.ascierto@gmail.com (P.A.A.); 3 Dipartimento di Neuroscienze e Scienze Riproduttive ed Odontostomatologiche, Università degli Studi di Napoli “Federico II”, 80040 Naples, Italy; 4 Immuno-Oncology & Targeted Cancer Biotherapies, University of Sassari, 07100 Sassari, Italy

**Keywords:** melanoma, tumor-infiltrating lymphocytes, tumor-associated antigens, mRNA-based therapy, immune checkpoint inhibitors, prognosis

## Abstract

Messenger RNA (mRNA) vaccines are emerging as promising tools capable of reshaping how cancer interacts with the immune system and responds to immunotherapy. These vaccines not only act as platforms for antigen delivery but can also influence the tumor microenvironment (TME), fostering a shift from immunologically “cold’’ conditions toward “hotter’’ and treatment-responsive states. In melanoma, this capability has been found to enhance the efficacy of the immune checkpoint inhibitors (ICIs), as mRNA-based priming can provide the robust antitumor activation needed for more effective checkpoint blockade. Early clinical studies with personalized or off-the-shelf vaccines showed benefits in patients with high-risk resected melanoma or refractory to PD-1 inhibition. Combining mRNA vaccines with ICIs, along with other immunomodulatory strategies, may be helpful to overcome resistance arising from the TME and achieve more durable clinical benefits. Besides these advances, computational and in silico modeling are providing new insights into how mRNA vaccines modulate the TME, helping to identify factors such as antigen-presenting cell (APC) density, CD8^+^ T-cell infiltration, and macrophage polarization that may predict treatment success and guide personalized strategies. Together, these developments indicate that combining mRNA vaccination with ICIs, supported by computational tools, may improve clinical outcomes in melanoma and, potentially, in selected tumor types with favorable immunological features, although important biological constraints limit direct extrapolation to less immunogenic malignancies.

## 1. Introduction

Cancer vaccines are designed to stimulate the immune system, thereby enabling the control of tumor growth and the eradication of cancer cells. Among the various vaccine technologies, mRNA-based cancer vaccines exhibit exceptional advantages, positioning them as a particularly helpful approach in oncological immunotherapy.

As mRNA-based vaccines have emerged as a powerful tool in cancer immunotherapy due to their versatility and ability to elicit robust antigen-specific immune responses, melanoma has notably served as one of the first solid tumors in which these vaccines were clinically explored, paving the way for their broader application in oncology [1].

Cutaneous melanoma is an aggressive and potentially lethal neoplasm arising from the malignant transformation of melanocytes, specialized cells that produce the pigment melanin, located primarily in the basal layer of the epidermis [2]. Over the past few decades, the progressive increase in the incidence of cutaneous melanoma has made it a growing global health concern [3,4]. It is worth emphasizing that, when diagnosed in the early stage, the localized lesions can be successfully removed by surgical excision, and almost all melanomas are cured by this treatment. However, delayed diagnosis or detection failure may lead to a rapid transition of primary melanoma to a proliferative phase that results in invasion of the dermis and then metastases. Despite representing a small fraction of all skin cancers, melanoma accounts for most of the skin cancer-related mortality owing to its high metastatic potential. Moreover, due to the resistance to conventional therapies and the ability to evade immune surveillance, the management of advanced or metastatic disease remains challenging. Before the advent of systemic biological treatments—targeted therapy and immunotherapy with immune checkpoint inhibitors (ICIs)—advanced melanoma was historically associated with a dismal prognosis, with 5-year survival rates much below 10% [5,6].

The exploration of the molecular landscape underlying melanoma development and progression has led to the discovery of crucial genetic alterations [7]. The most common oncogenes involved in melanomagenesis are *BRAF* and *NRAS*. Mutations in the *BRAF* gene, primarily the variants at the V600 codon, occur in approximately 50% of melanomas and drive constitutive activation of the mitogen-activated protein kinase (MAPK) signaling pathway, promoting uncontrolled proliferation. Mutations in the *NRAS* gene, most commonly affecting codon 61, occur in approximately 20–25% of melanomas, are mutually exclusive with *BRAF* mutations, and result in persistent activation of the MAPK pathway primarily, as well as of the PI3K/AKT signaling cascade, contributing to cell survival and tumor growth [8,9]. Unlike *BRAF*-mutant melanomas, *NRAS*-mutant tumors lack effective targeted therapies, and current treatment strategies rely primarily on immunotherapy-based approaches [10]. In melanoma patients carrying *BRAF* V600 mutations, the development of selective BRAF inhibitors (e.g., vemurafenib, dabrafenib, and encorafenib) and MEK inhibitors (e.g., cobimetinib, trametinib, and binimetinib) has led to rapid tumor regressions and improved progression-free survival [11]. Unfortunately, most patients undergoing BRAF/MEK inhibitors relapse within 12–18 months due to the occurrence of resistance mechanisms, such as pathway reactivation or activation of compensatory signaling cascades [12,13].

In parallel, therapy with ICIs targeting CTLA-4 (e.g., ipilimumab) and PD-1/PD-L1 (e.g., nivolumab, pembrolizumab) has revolutionized the treatment of metastatic melanoma, introducing significant improvements in clinical outcome. Checkpoint immunotherapies are suitable for patients with both *BRAF*-mutant and *BRAF* wild-type melanomas, and although they present slower starting activity than targeted therapies, they have the potential to achieve long-term benefit and possibly lead many patients to remission [14,15]. The counterpart of ICI-based treatments is represented by immune-related adverse events.

The high immunogenicity of melanoma is potentially immune-response provoking, but melanoma cells can evade immune surveillance in different ways, such as downregulation or loss of MHC class I molecules, recruitment of immunosuppressive cell populations, and upregulation of inhibitory immune checkpoints. In this scenario, ICIs work against tumor-induced suppression through the blockade of inhibitory pathways and the reactivation of cytotoxic T lymphocytes, enabling them to recognize and eliminate tumor cells more effectively, thereby enhancing the overall antitumor immune response. Not all patients respond to ICIs, urging the need for further therapeutic approaches [16,17,18,19]. Owing to its high tumor mutation burden (TMB), which increases the likelihood of producing neoantigens capable of triggering robust T-cell responses, melanoma remains a leading candidate for immunotherapy. This intrinsic immunogenicity has renewed interest in therapeutic cancer vaccine strategies aimed at further enhancing antigen-specific immunity against tumor-derived targets.

Although earlier approaches employing peptides, whole cells, dendritic cells, or viral vectors produced variably disappointing clinical outcomes, recent technological advancements have reinvigorated the field [20]. Personalized mRNA-based vaccines and tumor-infiltrating lymphocyte (TIL) therapies are emerging as next-generation immunotherapeutic modalities for melanoma [21,22]. Moderna’s mRNA-4157/V940 encodes patient-specific neoantigens derived from individual tumor mutations, enabling a tailored immune response with minimal collateral damage to healthy tissue [23]. Notably, mRNA-4157/V940 has shown promising results in combination with pembrolizumab in patients with resected high-risk melanoma [23,24]. In parallel, TIL therapy, which involves isolating, expanding, and reinfusing autologous lymphocytes from the tumor microenvironment (TME), has gained significant traction, especially following the recent FDA approval of Lifileucel [25]. These strategies exemplify the ongoing shift toward individualized treatment paradigms in advanced melanoma and are under active investigation as stand-alone therapies or in combination with other immunotherapeutic modalities. The high TMB rate of melanoma provides a vast repertoire of immunogenic epitopes suitable for mRNA-based targeting. Preclinical and early clinical studies have demonstrated that these vaccines can induce robust antigen-specific immunity, remodel the immunosuppressive TME, and potentially enhance the efficacy of ICIs by converting “cold” tumors into “hot” ones.

mRNA vaccine technology, which was prompted by the COVID-19 pandemic, has significantly evolved over the years, and several mRNA vaccine platforms have been developed and validated in preclinical studies [26]. Advances in RNA sequence engineering have improved the translational efficiency of synthetic mRNA, and a wide spectrum of highly efficient and non-toxic RNA carriers has been developed, allowing prolonged antigen expression in vivo. Furthermore, the main key advantages include rapid and scalable production, high flexibility in antigen design, non-integration into the host genome, and intrinsic adjuvanticity [27,28].

Significant obstacles persist: the optimization of delivery systems such as lipid nanoparticles (LNPs), the enhancement of mRNA stability and translation efficiency, and, not least, the persistent need to overcome immune tolerance mechanisms. The immunosuppressive milieu of the TME, characterized by elevated levels of TGF-β, IL-10, and metabolic suppressive factors like adenosine and IDO1, can dampen vaccine-induced responses. Strategies to address these barriers include the co-delivery of immunostimulatory adjuvants, combination with checkpoint inhibitors, and engineering of mRNA constructs to include cytokines or costimulatory molecules [29,30].

This review aims to explore how deeply mRNA vaccines can reshape the tumor–immune interface and enhance responsiveness to ICIs in the context of melanoma, and to define the current landscape and future directions of mRNA-based cancer vaccines as transformative agents in melanoma immunotherapy.

## 2. Methods of Literature Search

We searched PubMed/MEDLINE, Web of Science, and clinicaltrials.gov in the last five years up to January 2026 using the following terms: “melanoma,” “mRNA vaccine”, “tumor immunology”, “immune checkpoint inhibitors”, “tumor microenvironment”, “tumor-infiltrating lymphocytes”, “neoantigens”, “tumor associated antigens”, “TME remodeling”, and “computational biology”. We mainly included preclinical and (early) clinical studies, as well as reports on hypothesis/in silico investigations.

Original research studies and review articles were screened, whereas non-peer-reviewed reports and publications in languages other than English were excluded. We also reviewed the reference lists of key papers to identify additional primary sources.

This review was carried out as a narrative synthesis rather than a systematic review. We did not follow a PRISMA-style study selection process with formal record counts, nor did we conduct a risk-of-bias assessment. We summarize the findings qualitatively, focusing on evidence that is consistent across biological, translational, and clinical studies.

## 3. Mechanisms of Action of mRNA Vaccines in Melanoma and Their Impact on TME

mRNA-based vaccines have emerged as an innovative opportunity in the therapeutic landscape of melanoma, offering a highly flexible and targeted approach that allows one to elicit robust and specific anti-tumor immune responses and eventually spare normal tissue cells. Unlike traditional vaccines or DNA-based platforms, mRNA vaccines operate in the cytoplasm, avoiding genomic integration and allowing for transient, yet efficient, expression of immunogenic proteins [31].

**Figure 1:**
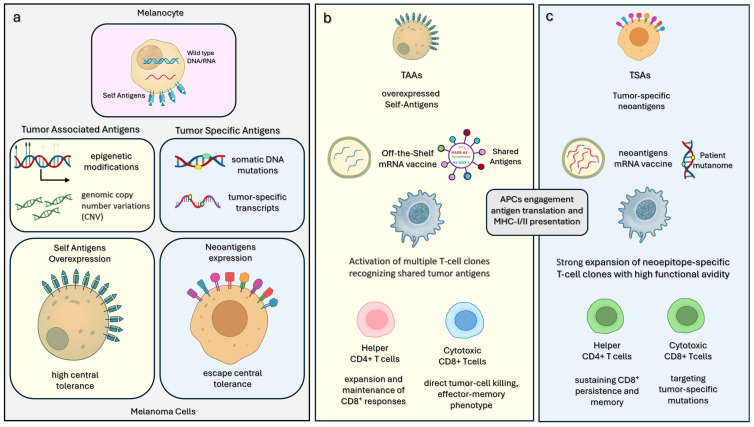
Tumor-Associated Antigens (TAAs) and Tumor-Specific Antigens (TSAs) as distinct targets for mRNA vaccine platforms in melanoma. (**a**) TAAs are unmutated self-antigens pathologically overexpressed via epigenetic dysregulation and genomic amplifications, with central tolerance limiting their immunogenicity. TSAs represent neoantigens originating from patient-specific somatic alterations, including non-synonymous SNVs, indels, and gene fusions; as neoepitopes, they intrinsically bypass central tolerance, eliciting de novo, high-affinity immune responses. (**b**) TAAs and Off-the-Shelf Vaccines target shared antigens prevalent across different patients and stimulate multiple T-cell clones, inducing an effector-memory phenotype and promoting direct tumor-cell killing by cytotoxic CD8^+^ T-cells. (**c**) TSAs and Personalized Vaccines target unique patient-specific alterations, triggering a strong expansion of neoepitope-specific T-cell clones with high functional avidity, where CD4^+^ helper T-cells play a crucial role in sustaining CD8^+^ persistence and memory. The Figure represents a conceptual model derived from the evidence reported in the text.

According to the nature of the encoded product, mRNA vaccines are currently grouped into categories encompassing tumor antigens, cytokines, antibodies, and immunomodulatory receptors [32,33]. The optimal target should be an antigen that exhibits both immunogenicity and specificity to cancer cells, so the identification and characterization of tumor-associated antigens (TAAs) and tumor-specific antigens (TSAs) have opened the avenue not only to better understand melanoma progression and differentiation but also to rely on suitable and more effective therapeutic strategies (Figure 1) [34].

The TSAs act as neoantigens, being mainly derived from mutations that occur in tumor DNA and are specifically expressed in tumor cells. The major advantage of mRNA vaccine platforms lies in their flexibility and customizability, being mRNAs engineered to encode shared melanoma-associated antigens or personalized neoantigens derived from non-synonymous somatic mutations unique to an individual’s tumor [28,35].

Next-generation sequencing (NGS) approaches and advanced immunoinformatic tools have enabled the development of highly personalized strategies for the identification of immunogenic neoepitopes with predicted affinity for autologous HLA molecules. These neoepitopes constitute the molecular basis of personalized cancer vaccines (PCVs), which are designed to induce selective cytotoxic T-cell responses against tumor-specific antigens, thereby optimizing therapeutic efficacy while minimizing collateral toxicity [36]. In this context, the clinical efficiency of the immune response largely depends on the delivery system used to deliver mRNA molecules. This is a challenging field, and various delivery systems have been explored to overcome the intrinsic limitation of the mRNA itself and, at the same time, enhance its efficacy. Among these, lipid nanoparticles (LNPs) are so far the most popular and first option for mRNA delivery, and a growing number of mRNA-LNP cancer vaccines are currently in clinical trials [37,38].

Following administration, LNPs protect mRNA from enzymatic degradation and facilitate its uptake by antigen-presenting cells (APCs), particularly dendritic cells (DCs). Once internalized, the mRNA is translated into tumor antigens, which are processed and presented on both MHC class I and II molecules. This dual presentation pathway enables the activation of CD8^+^ cytotoxic T lymphocytes and CD4^+^ helper T-cells, orchestrating a coordinated antitumor immune response [27]. Activated CD8^+^ T-cells specifically target and induce apoptosis in melanoma cells that have the same neoantigen by which the lymphocyte was primed. CD4^+^ helper T-cells release cytokines and chemoattractants, amplifying the cytotoxic immune response of CD8^+^ T-cells and recruiting other immune cells.

In addition to antigen presentation, mRNA molecules can act as innate immune stimulants by engaging pattern recognition receptors (PRRs), such as Toll-like receptors (TLR3, TLR7/8), leading to the production of type I interferons and pro-inflammatory cytokines. These signals promote DC maturation and enhance T-cell priming, further amplifying the adaptive immune response [39]. The LNPs used for mRNA delivery are not simply passive carriers; certain ionizable lipids within these formulations have been shown to activate innate immune pathways, including complement activation and pro-inflammatory cytokine release, thereby contributing to APCs recruitment and enhancing the immunogenicity of mRNA vaccines [40]. Recent advances have enabled the rational design of mRNA constructs that incorporate adjuvant-like features directly into the vaccine payload. For example, incorporating sequences encoding immunostimulatory cytokines or co-stimulatory ligands can further modulate the TME and promote a shift toward a pro-inflammatory, immune-permissive state [39,41,42]. This multifunctional design allows mRNA vaccines to act not only as antigen delivery systems but also as active immune modulators, capable of reshaping both systemic and local immune responses.

Preclinical studies in melanoma models have further demonstrated that mRNA vaccines induce potent antigen-specific responses, leading to epitope spreading and broadening immune recognition across multiple tumor antigens [21,43]. Moreover, mRNA vaccination enhances the infiltration of cytotoxic T-cells into the TME and reduces immunosuppressive populations such as regulatory T-cells (Tregs) and myeloid-derived suppressor cells (MDSCs), contributing to a more immunostimulatory milieu [42]. Beyond their molecular mechanisms, mRNA vaccines exert profound effects on the tumor microenvironment, reshaping immune cell composition and function.

## 4. Intracellular Alterations as Players for TME Status Modifications

**Figure 2:**
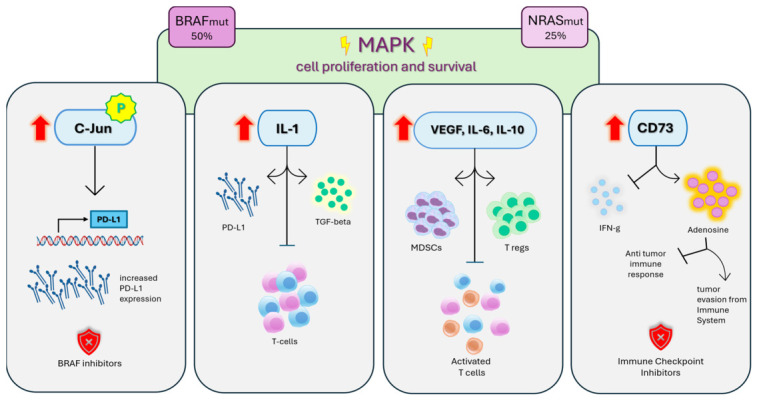
Impact of MAPK pathway activation on the melanoma TME. Oncogenic signaling triggered by *BRAF* or *NRAS* mutations promotes an immunosuppressive milieu: the c-Jun-mediated upregulation of PD-L1, the secretion of inhibitory cytokines such as IL-1, IL-6, and IL-10, and the production of VEGF promote the recruitment of MDSCs and T-regs. Additionally, increased CD73 expression leads to the accumulation of adenosine, further suppressing anti-tumor immune responses and contributing to resistance against immune checkpoint inhibitors. The Figure represents a conceptual model derived from the evidence reported in the text.

In melanoma, the activation of the NRAS-BRAF-MEK-ERK (known as the mitogen-activated protein kinase, MAPK) signal transduction pathway plays a central role in upregulating cell proliferation and survival. The melanoma cells carrying activated MAPK pathway—mostly through oncogenic mutations in *BRAF* (about 50% of cases) and *NRAS* (about 25% of cases) genes, which are mutually exclusive and usually non-overlapping in the same patients—may present additional molecular alterations that have been demonstrated to interfere with the response to ICI-based immunotherapy (Figure 2). In particular, the oncogenically activated MAPK pathway has been associated with:

- Activation of c-Jun that regulates a wide range of cellular processes, including cell proliferation, differentiation, survival, apoptosis, and inflammation [44]. Active c-Jun transcriptionally modulates the PD-L1 promoter activity at the nuclear level and contributes to increasing its expression [45]. Interestingly, the PD-L1 overexpression mediated by the activated c-Jun was also found to determine resistance to BRAF inhibitors, further confirming the tight interaction between immune regulation mechanisms and molecular components controlling cell proliferation and survival [45].
- Increased secretion of interleukin-1 (IL-1) that, in turn, may upregulate expression of PD-L1 and immunosuppressive cytokines, such as TGF-beta, thus promoting T lymphocyte suppression [46,47].
- Reduced expression of tumor-associated antigens helps melanoma cells to escape adequate recognition by the immune system [48].
- Increased expression of VEGF and immunomodulatory cytokines like IL-6 and IL-10, capable of promoting both the accumulation of immunosuppressive cells, such as MDSCs and Tregs, and depletion of activated T-cells into the TME [49].
- Increased expression of CD73, which in turn promotes the conversion of the extracellular ATP into adenosine and induces its accumulation into the TME [50,51]. Adenosine acts as an immunosuppressive metabolite that contributes to tumor evasion from the immune system [50]. High levels of CD73-induced adenosine inhibit multiple immune effectors, including CD8^+^ T-cells, natural killer cells, and dendritic cells (DCs), while enhancing immunosuppressive TME components, such as T-regs and MDSCs, and lowering the rates of the interferon-gamma (IFN-γ) signature, thus contributing to resistance to checkpoint inhibition [52].

Overall, all molecular modifications induced by MAPK activation are aimed at creating a tumor microenvironment unfavorable for the antitumor activity of the immune system in melanoma. As a confirmation of this, melanoma patients treated with BRAF and MEK inhibitors have been demonstrated to present enhancement of the expression of melanoma antigens and immune stimulatory cytokines, depletion of immunosuppressive cell populations (including tumor-associated macrophages (TAMs) and T regulatory cells (T-regs)), downregulation of immunosuppressive cytokines (including CD73), and increased CD8^+^ T-cell infiltration into the TME [53]. Further confirming this, melanomas progressed after treatment with BRAF/MEK inhibitors were associated with a decrease in CD8^+^ T-cell infiltration and/or an increase in immunosuppressive T-regs [54]. In addition to the above-described modifications responsible for reversion of the TME immunosuppression, inhibition of the activated MAPK pathway was found to promote PD-L1 overexpression in both preclinical models and metastatic melanoma patients [49,55].

This latter evidence represented the rationale for combining treatments with ICIs aimed at blocking the PD-1/PD-L1 axis and BRAF/MEK inhibitors [56]. A phase III study evaluated treatment with vemurafenib + cobimetinib ± atezolizumab in 514 patients with advanced *BRAF*^V600E^-mutant melanomas [57]. The median progression-free survival (PFS) was 15.1 months vs. 10.6 months in the experimental vs. control arm, respectively (HR: 0.78; 95% CI: 0.63–0.97). The estimated 2-year overall survival (OS) was 60% in the atezolizumab arm vs. 53% in the control arm. Another phase III COMBI-I trial (dabrafenib + trametinib ± spartalizumab) randomized 532 patients with advanced *BRAF*^V600E^-mutant melanomas [58]. The median PFS was 16.2 vs. 12.0 months in the spartalizumab arm vs. the control arm (HR: 0.82; 95% CI: 0.66–1.039). The estimated 2-year OS was 68% vs. 62% in the experimental vs. control arm, respectively. Finally, a phase II study randomized 120 patients with advanced melanoma and a *BRAF*^V600E/K^ mutation to receive dabrafenib + trametinib ± pembrolizumab [59]. The median PFS was 16.9 vs. 10.6 months in the experimental vs. control arm. The OS at 24 months was 63% vs. 51.7% in the experimental vs. control arm, respectively. In front of high toxicity (overall, grade ≥ 3 treatment-related adverse events were recorded in >50% of cases within the three studies), the efficacy of the triple combinations was disappointing. This suggests that simultaneously targeting the increased expression of PD-L1 induced by the BRAF/MEK inhibition does not offer an advantage in improving the clinical outcome (unless better patient selection is performed in the future).

**Figure 3:**
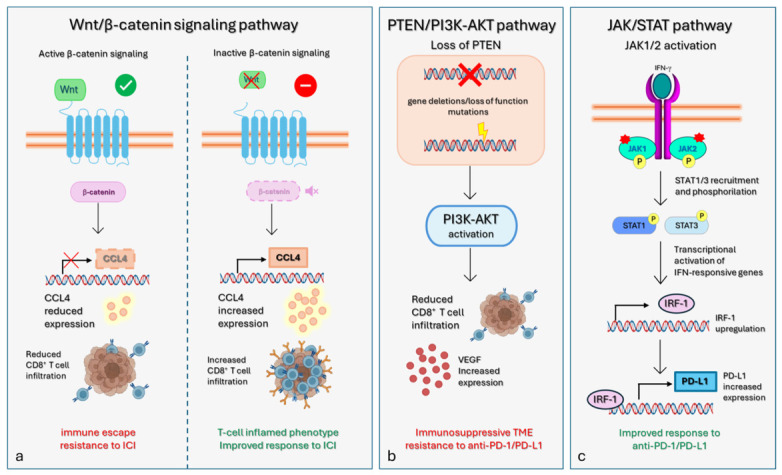
Additional intracellular pathways affecting TME, immune escape, and therapy response in melanoma. (**a**) Activation of the Wnt/β-catenin signaling pathway leads to CCL4 silencing, which impairs T-cell priming and results in a non-inflamed phenotype, whereas loss of β-catenin restores a T-cell-inflamed phenotype; (**b**) PTEN loss activates the PI3K-AKT pathway, reducing CD8^+^ T-cell infiltration and increasing the expression of immunosuppressive factors like VEGF; (**c**) upregulation of the JAK/STAT pathway, particularly through IRF-1, increases PD-L1 expression and enhances the tumor’s immunogenic profile, which has been directly correlated with better PFS and an improved response to anti-PD-1/PD-L1 therapy. The Figure represents a conceptual model derived from the evidence reported in the text.

In addition to the MAPK oncogenic activation, intracellular alterations in other distinct molecular pathways may interfere with the TME immune activity (Figure 3):

- Activation of the Wnt/β-catenin signaling pathway has been associated with the induction of immunotolerance through transcriptional silencing of the CCL4 gene; the reduction in CCL4 levels in the TME contributes to the impairment of the priming of antitumor T-cells, whereas silencing of β-catenin restores CCL4 production, leading to increased expression of PD-L1 and higher density of CD8^+^ T-cells [60]. In other words, inactive β-catenin signaling is associated with a T-cell-inflamed phenotype, while a constitutively active β-catenin signaling is associated with poor T-cell infiltration, immune escape, and resistance to immunotherapy [61,62]. Activation of the β-catenin pathway was also demonstrated to contribute to improving the outcome of *BRAF*-mutated melanoma patients treated with MAPK inhibitors, with the longest survivals achieved in patients showing a high density of CD8^+^ T-cells and low expression of β-catenin [63].
- Loss of PTEN protein, which is secondary to gene deletions and loss-of-function mutations, leads to activation of the PI3K-AKT pathway and has been associated with decreased infiltration by CD8^+^ T-cells in the TME of metastatic melanoma, as well as resistance to anti-PD-1/PD-L1 treatments [62,64]. Silencing of *PTEN* was also found to induce the expression of some immunosuppressive cytokines, particularly VEGF [64]. A high prevalence of *PTEN* inactivation has been observed in the progression of melanoma to the brain [65,66,67,68], allowing us to speculate that the occurrence of such an alteration may hamper the response to the immune checkpoint blockade with anti-PD-1/PD-L1 agents in mono-immunotherapy in melanoma brain metastases [62,68]. In this regard, it should be noted that the combination of immune checkpoint inhibitors (nivolumab and ipilimumab) seems to overcome the immunosuppressive effects linked to *PTEN* silencing and can generate a higher response rate in brain metastases [69,70,71].
- Upregulation of the JAK/STAT pathway has been associated with both increased PD-L1 expression and interaction with IFN-gamma-dependent signaling [72]. Activation of *JAK1* and *JAK2*—mostly by the acquisition of deleterious gene mutations—induces phosphorylation and nuclear translocation of the signal transducer and activator of transcription 1 (STAT1) and 3 (STAT3), with subsequent transcriptional activation of interferon-responsive genes [73]. Among upregulated genes, *interferon regulatory factor 1* (*IRF-1*) mainly acts on the PD-L1 promoter, thus increasing its expression, and genes underlying the antigen presentation machinery contribute to increasing the melanoma cell immunogenicity [72,73]. High levels of nuclear IRF1 in melanoma cells correlate with better PFS in patients treated with anti-PD-1 therapy [74]. At the same time, binding of IFN-γ to the interferon-gamma receptor (IFNGR) protein complex induces cell cycle arrest in melanoma cells through upregulation of the JAK/STAT pathway again, which has been found to also promote the direct inhibition of the proliferation enhancer cyclin-dependent kinase 6 (CDK6) and, indirectly, accumulation of the cyclin-dependent kinase inhibitor p27 [72]. Recently, our group found that advanced melanoma patients with concurrent *BRAF* and *JAK1/2* mutations presented a significantly favorable outcome when treated with the combination of nivolumab and ipilimumab in a first-line setting [75,76]. These findings appear to conflict with the role initially given to *JAK1/2* mutations that had been associated with resistance to anti-PD-1 therapy in melanoma [77,78]. However, *JAK* mutations have been evaluated at different times in two distinct series of patients’ treatments: at the time of resistance onset after a mono-immunotherapy with anti-PD-1 [77,78] and at baseline before the administration of a combination of anti-PD-1 and anti-CTLA-4 [75]. As a confirmation of this latter role, responses to anti-PD-1 have also been reported in patients with other *JAK*-mutant malignancies, such as colorectal cancers [45] and hematological neoplasms [79,80].

## 5. Remodeling the Tumor Microenvironment: From Immune Desert to Immune Activation

In melanoma, the tumor microenvironment plays a critical role in shaping disease progression and therapeutic outcomes. The TME consists of a highly structured and dynamic ecosystem within neoplastic tissues, encompassing a broad spectrum of cellular and acellular components. These include cancer cells and various non-malignant cell types, such as infiltrating immune populations, cancer-associated fibroblasts (CAFs), endothelial cells (ECs), extracellular matrix (ECM) constituents, and a complex network of soluble factors such as cytokines and chemokines [81].

Based on the presence and spatial distribution of tumor-infiltrating lymphocytes (TILs) and inflammatory signals, tumors are commonly classified into immune-inflamed, immune-excluded, and immune-desert phenotypes. The immune-inflamed phenotype is characterized by the presence of both CD4- and CD8-expressing T-cells, as well as proinflammatory cytokines providing a more favorable environment for T-cell activation and expansion, including type I and type II IFNs, IL-12, IL-23, IL-1β, tumor-necrosis factor (TNF)-α, and IL-2 [82,83]. The immune-excluded phenotype is also characterized by the presence of abundant immune cells, which are, however, retained in the stroma and do not penetrate the parenchyma. The immune-desert phenotype is characterized by a paucity of T-cells in either the parenchyma or the stroma of the tumor. Although myeloid cells may be present, the general feature of this profile is the presence of a non-inflamed TME with few or no CD8-carrying T-cells. Immune-desert tumors are particularly resistant to ICIs due to the absence of pre-existing antitumor immunity. The immune-desert and immune-excluded phenotypes can be considered as non-inflamed tumors.

Non-inflamed tumors typically secrete cytokines that promote immune tolerance or suppress immune responses. Their microenvironment is enriched in immunosuppressive or homeostasis-maintaining cell populations, including T-regs, MDSCs, and TAMs. Notably, T-regs are not confined to non-inflamed tumors, as they frequently coexist with effector T-cells at inflammatory sites, where they play a critical role in preserving immune homeostasis even in the context of active antitumor immunity [84].

Evidence from preclinical tumor models suggests that mRNA-based cancer vaccines can influence the TME, although these effects appear highly context dependent and shaped by variables such as tumor type, vaccine formulation, and treatment setting [85]. The expression of TAAs has been reported to enhance the priming and activation of CD4^+^ and CD8^+^ T-cells. Antigen-driven immune stimulation is commonly accompanied by increased production of pro-inflammatory cytokines and chemokines, which contribute to shaping the inflammatory milieu of the TME. These cytokine and chemokine signals may facilitate the recruitment and infiltration of effector immune populations into the tumor site, thereby contributing to increased immune activity within the TME. mRNA cancer vaccines have also been reported to activate dendritic cells and other antigen-presenting cells, including macrophages and B cells, at least in part through innate immune signaling pathways, thereby supporting tumor antigen presentation to T-cells and reinforcing antigen-specific antitumor immune responses. In addition, changes in innate immune activation induced by mRNA-based vaccination have been reported to be associated with modulation of tumor-associated macrophage polarization in selected preclinical models, with shifts from immunosuppressive M2 macrophages toward more pro-inflammatory M1 macrophages [86]. A tendency toward a shift from M2-like to M1-like macrophages was previously reported in cell lines from other cancer types [87].

In many solid tumors, including melanoma, the TME commonly exhibits a substantial enrichment of M2 macrophages, associated with immune evasion and resistance to treatment [88,89]. An increasing use of computational biology is being observed to support cancer immunotherapy research, particularly in the context of TME remodeling [90]. Mathematical and computational models have also been applied to investigate immune cell behavior within the TME in mRNA-based cancer vaccination settings. As shown by Voutouri et al. [91], mechanistic mathematical models allow the simulation of complex tumor–immune interactions to explore how tumors and the immune system interact under different conditions. In melanoma models, simulations suggest that reducing extracellular matrix (ECM) density and maintaining intermediate cytokine levels may enhance CD8^+^ T-cell infiltration and improve vaccine distribution within tumors. These findings are primarily derived from in silico and preclinical modeling frameworks and remain largely predictive rather than experimentally validated in clinical settings [92]. While computational modeling provides a valuable framework for hypothesis generation and mechanistic exploration, its application to personalized vaccine strategies requires integration with tumor/normal sequencing, robust neoantigen prioritization pipelines, HLA typing, and feasible manufacturing timelines [43]. At present, its role should be considered supportive rather than directly actionable in clinical decision-making.

## 6. Synergy Between mRNA Vaccines and ICIs

mRNA vaccines—typically delivered through LNPs—largely enter the cytoplasm of APCs [93]. At the intracellular level, mRNAs are heavily translated into the determined neoantigen proteins. Therefore, neoantigen production is exclusively endogenous, which represents a powerful immunological advantage. Indeed, endogenously synthesized proteins are processed by the proteasome into short peptides, which are then combined with MHC class I molecules within the endoplasmic reticulum. The peptide-MHC-I complex is then transported to the cell surface [93].

The mRNA vaccines, by eliciting robust neoantigen-specific T-cell responses, offer a valid complement to ICIs [94]. At the same time, ICIs preserve the functionality of vaccine-induced T-cells, preventing exhaustion and promoting their persistence in the tumor microenvironment [95]. Targeting PD-1/PD-L1 and CTLA-4 has revolutionized treatment paradigms, yet a significant proportion of patients still experience primary resistance or relapse after an initial response [96]. PD-L1 expression, TMB, and transcriptomic signatures have been extensively evaluated as predictors of response to immune checkpoint blockade [97]. Despite overexpressed PD-L1 or high TMB rates being associated with improved clinical outcomes, their ability to distinguish responders from non-responders remains limited, as durable responses also occur in contexts lacking such features [98]. Compared to TMB, which just quantifies the mutational load, neoantigen burden could likely represent a more biologically informative metric.

In addition to high levels of PD-L1 and TMB/neoantigens, we are far from using a biomarker in clinical practice that can accurately predict a benefit from ICI alone or combined with a vaccine, allowing us to only speculate that a response/resistance predictive algorithm could include other potentially effective baseline conditions: (a) presence and density levels of TILs into TME, though higher TIL rates do not necessarily classify the tumor as immunologically responsive since a variable part of tumor-specific CD8^+^ T-cells might be “exhausted” due to persistent exposition to antigenic stimulation [99]; (b) preserved interferon-gamma signature expression, which has been reported to be able to adequately differentiate patients who were likely to benefit from immunotherapy [100], though many IFN-γ scoring systems and gene-expression profiles (GEP) exist; (c) mutationally activated status of the JAK/STAT signaling pathway [75]; (d) impaired antigen presentation machinery (i.e., HLA expression changes, stromal contexts linked to immune exclusion) [101]; and (e) the TME level of immunosuppressive cells (i.e., preponderance of T-regs, MDSCs, and/or TAMs).

Preclinical studies in murine melanoma models showed that peptide-based vaccination targeting selected neoantigens could induce strong antitumor immunity, with notable efficacy in both prophylactic and therapeutic settings [102]. They demonstrated that passenger mutations, even when not oncogenic, can exert an effective stimulation of the antitumor immunity. However, not all synthetic neoantigens are immunogenic, underscoring the need for careful selection of candidates to ensure vaccine efficacy. These findings, along with others [103], pointed to the importance of considering both neoantigen selection and the quality of T-cell priming when designing effective vaccination strategies.

The quality of T-cell priming is critical for the success of immune checkpoint blockade. Verma et al. observed that PD-1 inhibition in sub-optimally primed CD8^+^ T-cells can paradoxically lead to the emergence of dysfunctional PD-1^+^CD38^hi populations, which are associated with poor effector function and resistance to anti-PD-1 therapy [104]. This observation highlighted a potential vulnerability in immunotherapy strategies: when ICIs are introduced before robust antigen-specific activation, they may amplify exhausted or ineffective T-cell states. Recent insights underscore that strategies specifically designed to broaden and enhance the quality of T-cell priming may mitigate this risk by promoting the expansion of highly activated, neoantigen-specific T-cell populations before PD-1 blockade [105]. By strengthening the initial immune landscape, these vaccines may not only prevent dysfunctional differentiation but also work synergistically with ICIs to achieve deeper and more durable antitumor responses.

Emerging translational research further supports this rationale [106]. Gao et al. [85] highlight how mRNA vaccines can modulate the TME by enhancing T-cell infiltration and reducing immunosuppressive cell populations; beyond enhancing response rates, mRNA vaccines may also mitigate immune escape by broadening the spectrum of tumor antigens recognized by the immune system.

Clinical evidence is beginning to mirror preclinical insights [107]. Clinical trials of mRNA cancer vaccines have demonstrated encouraging results in terms of safety, immunogenicity, and efficacy. Pioneering candidates, such as BioNTech’s BNT111 and Moderna’s mRNA-4157, have shown promising outcomes in targeting melanoma and solid tumors (see below). The first-in-human KEYNOTE-603 trial established the safety and immunogenicity of the personalized mRNA vaccine mRNA-4157 (V940), both as monotherapy and in combination with pembrolizumab [108], providing the basis for subsequent studies, such as the KEYNOTE-942 and Lipo-MERIT trials.

As detailed below, the KEYNOTE-942 study, a randomized phase II adjuvant trial in patients with resected stage III/IV melanoma, evaluated a personalized neoantigen mRNA vaccine (mRNA-4157; V940) in combination with the anti-PD-1 pembrolizumab and demonstrated a significant improvement in recurrence-free survival, corresponding to an approximately 49% reduction in the risk of recurrence or death, with a median follow-up of 3 years. Efficacy was consistent across subgroups, including patients with low PD-L1 expression and low tumor mutational burden, and was accompanied by improvement in distant metastasis-free survival. Moreover, vaccine-induced T-cell responses were positively correlated with clinical benefit [23,24].

In parallel, the Lipo-MERIT phase I trial evaluated the safety and tolerability of BNT111 (FixVac) in patients with stage III/IV unresectable melanoma alone or in combination with cemiplimab [109], showing robust CD4^+^ and CD8^+^ T-cell responses and early signs of clinical activity, both as monotherapy and in combination with cemiplimab. Unlike fully personalized vaccines, BNT111 is an “off-the-shelf” formulation encoding four melanoma-associated antigens (NY-ESO-1, MAGE-A3, tyrosinase, and TPTE), which are commonly expressed in advanced melanoma. The results highlight that vaccines based on shared antigens, even if less tailored to individual tumors, can still deliver clinical benefit and offer practical advantages in terms of scalability and accessibility.

**Figure 4:**
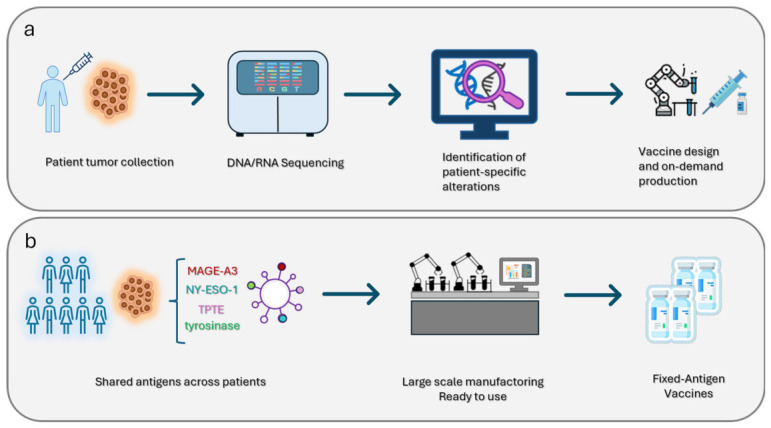
Comparative workflows for the development of individualized and fixed-antigen mRNA vaccine platforms. (**a**) Personalized Neoantigen Therapy: this workflow begins with the collection of patient tumor samples, followed by high-throughput DNA/RNA sequencing to identify unique somatic alterations. Advanced immunoinformatic algorithms are then utilized to select and prioritize immunogenic neoepitopes then utilized for on-demand vaccine design and production. (**b**) Off-the-Shelf Fixed-Antigen Vaccines: this strategy targets shared antigens (e.g., NY-ESO-1, MAGE-A3, tyrosinase, TPTE) that are commonly overexpressed across the melanoma patient population. This approach facilitates large-scale manufacturing and provides “ready-to-use” formulations, allowing for immediate clinical application without the need for patient-specific sequencing. The Figure represents a conceptual model derived from the evidence reported in the text.

Taken together, these two strategies represent complementary paths in mRNA vaccine development, balancing precision with scalability, with the aim to extend the benefits of cancer immunotherapy to a broader patient population (Figure 4).

Several translational challenges remain [110]: optimal strategies for antigen selection, vaccine scheduling, and biomarker-guided patient stratification are still under investigation. The integration of bioinformatics and machine learning enables improved antigen prediction, optimization of lipid nanoparticle formulations, and a better understanding of their interactions with immune cells [111]. Nevertheless, the convergence of mRNA vaccine technology with ICI therapy represents a rational and increasingly evidence-based approach to overcoming resistance and improving outcomes in melanoma.

While these results highlight the therapeutic potential of combining mRNA vaccines with immune checkpoint inhibitors, safety and feasibility considerations remain critical for clinical translation. Immune-related adverse events (irAEs) observed with combination strategies appear largely consistent with the known safety profiles of ICIs, with clinical data indicating manageable toxicity and no clear evidence of significant additive immune-related toxicity from mRNA vaccination [112,113,114]. Across multiple studies, mRNA-based cancer vaccines have been generally well tolerated, with most adverse events being of low-grade and transient. In addition, LNP-based delivery systems are associated with reactogenicity, including fever, fatigue, and injection-site reactions [115]. From a clinical standpoint, optimal integration with ICIs remains to be defined, including dosing schedules, sequencing (concurrent versus sequential), and treatment setting [112,113,114]. Emerging evidence suggests that adjuvant or minimal residual disease settings may represent the most favorable context for balancing efficacy and tolerability [116,117,118].

## 7. Clinical Evidence and Ongoing Trials in Melanoma

The development of cancer vaccines in melanoma has progressively evolved from early proof-of-concept studies demonstrating immunogenicity to more recent trials suggesting clinically meaningful—although still heterogeneous—signals of efficacy, particularly when combined with ICIs [107,108,111]. Historically, multiple platforms, including peptide-, dendritic cell-, and DNA-based vaccines, consistently induced antigen-specific immune responses without translating into durable survival benefits [107,108]. This discrepancy underscores a central limitation: effective vaccination requires not only immune activation but also the generation of functional tumor-specific T-cells capable of overcoming the immunosuppressive TME status [108,110].

A major inflection point has emerged with mRNA-based technologies, which integrate efficient antigen delivery with intrinsic adjuvanticity. By enabling endogenous antigen expression within APCs, mRNA vaccines promote coordinated MHC class I and II presentation, eliciting both CD8^+^ and CD4^+^ T-cell responses. In parallel, activation of innate immune pathways-particularly through type I interferon signaling-creates a pro-inflammatory context that enhances T-cell priming and synergizes with ICIs [107,109].

A critical question, however, is whether mRNA vaccines are intrinsically more immunogenic or whether their apparent superiority derives from their ability to reshape the immune context. Insight into this issue is provided by the SARS-CoV-2 mRNA vaccine experience. Administration of COVID-19 mRNA vaccines within approximately 100 days prior to ICI initiation has been associated with improved survival outcomes, with median overall survival increasing from 20.6 to 37.3 months (HR 0.51, 95% CI 0.37–0.71; *p* < 0.0001) [119]. Mechanistically, this effect appears to be mediated by systemic type I interferon induction, enhanced APC activation, and increased tumor PD-L1 expression, ultimately facilitating more effective T-cell priming and synergy with checkpoint blockade [119]. These findings suggest that the key contribution of mRNA-based strategies may lie in their capacity to reprogram the immune microenvironment rather than solely in antigen specificity. Within this evolving framework, two complementary strategies have emerged: personalized neoantigen vaccines and off-the-shelf shared-antigen approaches.

### 7.1. Immunological Differences Across Vaccine Modalities

The heterogeneity of vaccine platforms reflects fundamental differences in antigen presentation and immune activation [107,112]. mRNA vaccines currently represent the most advanced approach, combining intracellular antigen expression, dual MHC class I/II presentation, and intrinsic innate immune activation [107,109]. Lipid nanoparticle delivery further enhances lymphoid targeting and dendritic-cell uptake.

In contrast, peptide vaccines rely on exogenous antigen presentation and are limited by HLA restriction and immune tolerance, particularly when targeting shared tumor-associated antigens [108,120,121]. DNA vaccines are constrained by inefficient cellular uptake, whereas dendritic cell-based vaccines, although effective in antigen presentation, remain complex and have not consistently improved clinical outcomes [107,117,118].

### 7.2. Cancer Vaccines in Melanoma: mRNA and Peptide Platforms—Clinical Evidence

The mRNA vaccination strategies are diverging in two complementary approaches: personalized neoantigen vaccines and shared-antigen platforms, reflecting a balance between biological specificity and clinical feasibility.

Personalized mRNA vaccines such as V940 encode up to ~34 patient-specific neoantigens, enabling polyclonal T-cell responses that bypass central tolerance [43,108]. In the phase IIb KEYNOTE-942 trial, the combination of V940 with pembrolizumab significantly improved recurrence-free survival, reducing the risk of recurrence or death by 49% (2.5-year RFS: 74.8% vs. 55.6%) [23]. Despite these promising results, this approach is limited by manufacturing complexity and turnaround time, and its benefit appears most pronounced in minimal residual disease settings rather than advanced disease [107,113].

Off-the-shelf mRNA vaccines offer immediate availability and scalability. BNT111, encoding four melanoma-associated antigens (NY-ESO-1, MAGE-A3, tyrosinase, and TPTE), has demonstrated clinical activity in ICI-refractory melanoma, with objective responses (~18%) and disease control rates exceeding 50% [109,114]. These findings suggest that optimized delivery and strong innate immune activation can partially compensate for lower antigen specificity, supporting the feasibility of fixed-antigen approaches.

An alternative strategy focuses on targeting immunosuppressive pathways within the TME rather than tumor antigens alone. The IO102–IO103 peptide vaccine induces T-cell responses against IDO1 and PD-L1, aiming to dismantle dominant suppressive circuits. In the phase III IOB-013/KN-D18 trial, IO102–IO103 plus pembrolizumab improved median progression-free survival to 19.4 months compared with 11.0 months for pembrolizumab alone (HR 0.77, *p* = 0.0558), narrowly missing statistical significance [118]. Notably, a substantial benefit was observed in PD-L1-negative tumors (16.6 vs. 3.0 months; HR 0.54), suggesting activity in immunologically resistant disease [118]. These results can be interpreted as “near positive,” highlighting a clinically meaningful signal not fully captured by statistical thresholds.

The same targets have been explored using an mRNA platform (mRNA-4359). In checkpoint inhibitor-resistant melanoma, this strategy achieved a 67% objective response rate in PD-L1-positive patients, with evidence of antigen-specific T-cell expansion [122]. This approach combines the biological rationale of immune-modulatory targeting with the pharmacological advantages of mRNA delivery.

Notably, this strategy mirrors the conceptual framework of the IO102–IO103 peptide vaccine, which targets the same immunosuppressive pathways within the TME. However, the use of an mRNA platform may overcome several intrinsic limitations of peptide-based vaccination. While peptide vaccines rely on exogenous antigen presentation and are often constrained by HLA restriction and suboptimal cross-presentation, mRNA vaccines enable endogenous antigen expression within antigen-presenting cells, promoting more efficient MHC class I and II presentation and broader T-cell priming. In addition, mRNA formulations provide intrinsic adjuvanticity through innate immune activation, which may enhance both the magnitude and functional quality of T-cell responses.

Collectively, these observations support the concept that the effectiveness of cancer vaccines is determined not only by antigen selection but also by the capacity of the platform to induce robust and sustained immune priming.

Personalized vaccination strategies are now also moving into the first-line setting, with two conceptually distinct but complementary approaches represented by EVX-01 and INTerpath-012 [123,124]. EVX-01 is a personalized peptide-based neoantigen vaccine, generated through DNA/RNA sequencing and AI-guided selection of 7–10 patient-specific neoantigens. In a phase II study in previously untreated unresectable stage III/IV melanoma, EVX-01 plus pembrolizumab showed encouraging early activity, with a best objective response rate of 75% (12/16), a 54% conversion rate among evaluable patients, sustained responses at 24 months in 92% of responders, and 100% manufacturing success, together with vaccine-induced T-cell responses across all treated patients. Although derived from a small, single-arm cohort, these data support the feasibility of achieving clinically relevant immune responses with personalized peptide-based platforms.

In parallel, the phase II INTerpath-012 study extends the personalized mRNA neoantigen strategy of V940/mRNA-4157 into untreated advanced melanoma. This randomized, double-blind trial is evaluating intismeran autogene plus pembrolizumab versus placebo plus pembrolizumab in 160 patients with previously untreated unresectable stage III/IV cutaneous melanoma, with progression-free survival as the primary endpoint and ORR, duration of response, overall survival, and safety as secondary endpoints.

A similar paradigm extends to peptide-based approaches. Personalized peptide vaccines, such as EVX-01, maximize biological precision through neoantigen selection, whereas fixed peptide vaccines like IO102–IO103 prioritize accessibility and immune modulation of the TME. Taken together, these observations define a central trade-off in cancer vaccination between biological specificity and clinical practicality. Rather than representing competing strategies, personalized and fixed platforms may be viewed as complementary approaches, whose optimal integration—potentially guided by disease setting, tumor burden, and treatment timing—will likely determine the future trajectory of the field.

These differences highlight how mRNA platforms may overcome key limitations of peptide vaccines by enabling endogenous antigen expression and intrinsic adjuvanticity, thereby enhancing cross-presentation efficiency and promoting broader T-cell responses.

### 7.3. Platform Limitations and Evolving Strategies

Experience with earlier vaccine platforms provides essential context for current advances. Peptide vaccines can induce antigen-specific T-cell responses but are often limited by HLA restriction, suboptimal cross-presentation, and immune tolerance, resulting in modest clinical benefit [117,118,120,121]. Dendritic cell–based strategies improve antigen presentation but remain logistically complex and have not consistently translated into survival advantages [116,117]. These limitations are interpreted as platform-related rather than conceptual failures of vaccination. Insufficient innate immune activation and inefficient T-cell priming likely contributed to the limited efficacy of earlier approaches.

**Figure 5:**
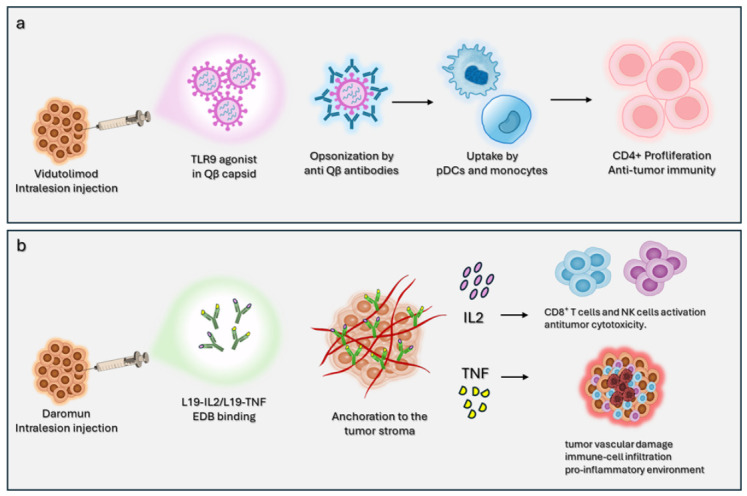
Mechanisms of action of intralesional neoadjuvant immunotherapies in melanoma. (**a**) Vidutolimod is a TLR9 agonist encapsulated in a Qβ capsid. After opsonization by anti-Qβ antibodies and uptake by pDCs and monocytes, it triggers CD4^+^ T-cell proliferation and orchestrates a robust systemic anti-tumor immunity, leading to high rates of major pathologic response (MPR). (**b**) Daromun is a combination of L19-IL2 and L19-TNF. These immuno-cytokines bind specifically to EDB-fibronectin in the tumor stroma, causing vascular damage and a pro-inflammatory TME. This enhances the recruitment of CD8^+^ T-cells and NK cells, promoting potent local cytotoxicity and improving recurrence-free survival. The Figure represents a conceptual model derived from the evidence reported in the text.

More recent strategies incorporating innate immune stimulation-such as TLR9 agonists or intralesional immunotherapies-highlight that effective vaccination requires both antigen delivery and modulation of the tumor microenvironment [125,126] (Figure 5).

### 7.4. Vaccines and ICIs: Beyond Priming

A consistent theme across clinical studies is the synergy between vaccines and ICIs. Vaccines provide the priming phase, generating tumor-specific T-cells, while ICIs enable their effector function [108,114]. Emerging data suggest that this interaction extends beyond simple priming and reflects broader immune reprogramming. Evidence from SARS-CoV-2 mRNA vaccination shows that systemic immune activation prior to ICI therapy can improve outcomes without increasing toxicity [119,127].

These findings support the concept that effective vaccines act not only as antigen delivery systems but also as modulators of the immune context, enhancing responsiveness to checkpoint blockade. This may explain the more consistent synergy observed with mRNA-based platforms compared with earlier peptide approaches.

### 7.5. Expanding Clinical Evidence Beyond Melanoma: Pancreas and Triple-Negative Breast Cancer

Clinical evidence about the therapeutic potential of mRNA-based cancer vaccines is emerging for other solid tumors.

In pancreatic ductal adenocarcinoma (PDAC)—a recognized non-immunogenic tumor—an individualized neoantigen mRNA vaccine (autogene cevumeran) demonstrated the ability to induce high-magnitude, de novo neoantigen-specific T-cell responses in approximately 50% of treated patients [128]. Vaccine responders exhibited prolonged recurrence-free survival compared with non-responders, suggesting a direct association between vaccine-induced T-cell expansion and clinical outcome. These findings are particularly relevant given the well-known resistance of PDAC to ICIs.

Similarly, in triple-negative breast cancer (TNBC), individualized mRNA neoantigen vaccines have shown the capacity to induce durable and polyfunctional T-cell responses targeting multiple patient-specific mutations [129]. In this study, nearly all patients developed vaccine-induced T-cell immunity, with persistence of functional responses for several years and a high proportion of patients remaining relapse-free at long-term follow-up [129]. Importantly, these responses included both effector and memory T-cell populations, suggesting the establishment of long-lasting immunological surveillance.

Taken together, these data extend the relevance of mRNA cancer vaccines beyond melanoma and strongly indicate that mRNA vaccination may be helpful in converting insufficiently primed or immunologically cold tumors into more treatment-responsive states. In other words, mRNA platforms appear well-suited to induce de novo antitumor immunity, supporting their broader application across tumor types and disease settings.

While these findings extend the relevance of mRNA cancer vaccines beyond melanoma, it is important to consider that melanoma represents a uniquely favorable biological context for this therapeutic approach. Cutaneous melanoma, particularly sun-exposed melanoma, is characterized by a high somatic mutational burden and UV-associated mutational signatures, which increase the probability of generating tumor neoantigens [130]. In contrast, other tumor types present distinct challenges. In NSCLC, although high TMB can associate with improved response to PD-1/PD-L1 blockade, pronounced intratumoral heterogeneity, increased production of subclonal neoantigens, and neoantigen-directed immune escape may limit the consistency of immune responses [131,132,133]. In colorectal cancer, limited neoantigen expression, poor T-cell priming, and reduced baseline immune infiltration contribute to lower responsiveness to ICIs [134,135].

In this sense, PDAC is characterized by a dense desmoplastic/stromal microenvironment and paucity or dysfunction of dendritic cells, which constrain antigen presentation and effective antitumor T-cell priming [136,137]. Across tumor types, successful implementation of mRNA vaccination likely depends on sufficient neoantigen quality and expression, intact antigen processing and presentation, the presence of functional cross-presenting dendritic cells, and preserved interferon-linked immune activation. Current evidence is limited by small sample sizes, early-phase trials, and heterogeneity in study design. Patient selection bias toward immunologically favorable populations and variability in neoantigen prediction and manufacturing further complicate interpretation. Larger randomized studies are needed.

A comprehensive overview of the key clinical trials discussed is provided in Table 1.

## 8. Conclusions

mRNA-based cancer vaccines are emerging as active modulators of the tumor–immune interface, not merely antigen-delivery tools. In the therapeutic landscape of melanoma, the main contribution of the mRNA vaccines appears to be the induction of robust T-cell priming together with immune-context reprogramming, thereby enhancing responsiveness to checkpoint blockade. Emerging clinical evidence provides encouraging indications that both personalized and off-the-shelf platforms can achieve clinically meaningful activity by extending the benefits of immunotherapy to a wider range of patients. Nonetheless, several practical questions remain, including which antigens should be prioritized and how vaccination should be integrated with checkpoint blockade, together with the challenges that come with combination therapy. Computational and in silico modeling offer useful tools in this direction, helping to clarify how specific features of the tumor microenvironment influence vaccine performance and supporting more tailored development pathways. With such clinical advancements, the growing role of mRNA vaccination within the current therapeutic landscape is becoming more evident, along with its potential to improve existing immunotherapy approaches. In this evolving scenario, mRNA-based vaccines are likely to become an increasingly integrated part of melanoma management, not simply as an addition but as a central component alongside targeted agents, checkpoint inhibition, and emerging immunomodulatory approaches, contributing to more flexible and patient-centered therapeutic options.

## Figures and Tables

**Table 1:** mRNA-based vaccine programs in cancers.

Trial	Tumor Type	Journal (Year)	Design	Endpoints	Key Results	Limitations	Status
Individualized neoantigen therapy mRNA-4157 (V940) plus pembrolizumab in resected melanoma (KEYNOTE-942)	Melanoma (stage III/IV resected)	J Clin Oncol (2024)	Phase II, randomized, adjuvant	RFS, DMFS, OS	HR ~0.51 (~49% risk reduction); improved DMFS; benefit across PD-L1-low and TMB-low	Phase II; interim follow-up; abstract-level data	Ongoing Phase III
Safety and immunogenicity of personalized neoantigen mRNA vaccine mRNA-4157 in combination with pembrolizumab (KEYNOTE-603)	Melanoma/solid tumors	Cancer Discovery (2024)	Phase I/II	Safety, immunogenicity, ORR	Strong neoantigen-specific CD8^+^ T-cell responses; early clinical activity	Small cohorts; non-randomized	Completed
A vaccine targeting multiple tumor-associated antigens induces polyfunctional CD4^+^ and CD8^+^ T cell responses in melanoma patients (BNT111)	Melanoma	Nature (2020)	Phase I	Safety, immunogenicity	Robust T-cell responses; signals of clinical activity	Early phase; small sample size	Ongoing
An individualized mRNA neoantigen vaccine for pancreatic ductal adenocarcinoma	PDAC	Nature (2023)	Phase I (adjuvant)	Safety, immunogenicity, RFS	~50% responders; prolonged RFS in responders	small sample size; selected population	Ongoing
Individualized neoantigen-specific mRNA vaccination induces durable T-cell responses in TNBC	TNBC	Nature Medicine (2023)	Phase I	Safety, immunogenicity	Durable polyfunctional T-cell responses	Early phase; limited cohort	Early-phase
mRNA-4359 encoding IDO1 and PD-L1 for advanced solid tumors	Solid tumors (PD-L1+)	Conference/early reports (2023–2024)	Phase I	Safety, ORR	Preliminary ORR signal in PD-L1+ resistant tumors	Very small cohorts; preliminary data	Ongoing
Personalized RNA mutanome vaccines mobilize poly-specific therapeutic immunity against cancer	Melanoma/solid tumors	Nature (2017)	Phase I	Safety, immunogenicity	Induction of CD4^+^ and CD8^+^ T-cell responses	Early phase; exploratory	Early-phase
Phase I study of mRNA-5671/V941 KRAS vaccine alone or with pembrolizumab	KRAS-mutant solid tumors	Clinical trial NCT 03948763	Phase I	Safety, immunogenicity	KRAS-specific T-cell responses (ongoing)	Clinical efficacy data not yet mature	Ongoing
Combination of IO102 and IO103 with immune checkpoint inhibitors in melanoma	Melanoma	Phase III (2023–2024)	Phase III, randomized	PFS, OS	Missed PFS significance; signal in PD-L1-negative	Platform limitations	Completed

Abbreviations: RFS, recurrence-free survival; DMFS, distant metastasis-free survival; OS, overall survival; ORR, objective response rate. Early-phase clinical development refers to phase I–II studies primarily designed to assess safety and immunogenicity. PDAC, pancreatic ductal adenocarcinoma; TNBC, triple-negative breast cancer.

## Data Availability

No new data were created in this study.
